# In the pursuit of perfect planning: comparison between Lightning Inverse Planning and GammaPlan Wizard for gamma knife radiosurgery

**DOI:** 10.2478/raon-2025-0039

**Published:** 2025-06-21

**Authors:** Victor Goulenko, Robert J Plunkett, Matthew B Podgorsak, Dheerendra Prasad

**Affiliations:** 1 Roswell Park Comprehensive Cancer Center, Radiation Medicine, Buffalo, NY, USA; 2 Roswell Park Comprehensive Cancer Center, Neurosurgery, Buffalo, NY, USA; 3 Department of Neurosurgery, Jacobs School of Medicine and Biomedical Sciences, Buffalo, NY, USA

**Keywords:** Gamma Knife, Wizard, Lightning, Inverse planning, Leksell GammaPlan

## Abstract

**Background:**

The Lightning^®^ software, was added to the Gamma Knife’s Leksell GammaPlan^®^ as a fully automated inverse planner, differently from the prior software, Wizard^®^. In this paper we compare their treatment planning capacity and quality.

**Materials and methods:**

Thirty-eight cases were compared under four different planning techniques. First, manual forward planning aided by the Wizard^®^ optimization tool. Second, inverse planning with Wizard^®^. The third and fourth plans used Lightning^®^ with and without consideration for organs at risk (OAR). They were analysed for: planning time, number of shots, coverage, selectivity, gradient index, bean-on time, and OAR dose. Comparison based on pathology was added due to their idiosyncrasies. For quality comparison, dose-volume histograms (DVH) were compared to plans developed under our treatment standards. Tumor’s volume and time to plan were correlated with Pearson’s coefficient.

**Results:**

Lightning^®^ had better coverage (8%) and gradient index (15%) but had 12% decrease in selectivity. Planning and delivery times had a reduction of 57% and 5% respectively, despite having three times the number of shots. Only Lightning^®^ with protection of OAR met the dose constrains in all plans. DVH showed similar plan qualities.

**Conclusions:**

Lightning^®^ allowed the planner to explore different optimization parameters to achieve a plan that suits the clinical problem at hand. It took less time to calculate shots placement, OAR protection and the ideal isodose line than the Wizard^®^. This can be useful to plan multiple and complex targets at a faster time, increase the patient’s tolerance and, may have a radiobiological advantage by impacting intra-fraction repair.

## Introduction

Planning for Gamma Knife Radiosurgery (GKRS) has evolved constantly since its inception in 1967 with improvement in imaging, advances in computing platforms and changes in collimator configuration with the release of new machine models. From Leksell GammaPlan^®^ (LGP) Version 5.34, plan optimization as well as shot (isocenter) placement was available as an option within LGP under the tradename Wizard^®^. The quality of a plan has largely depended on the skills and experience acquired by the planner. Inexperienced planners can find the learning curve for forward planning steep, especially when risk structures, eloquent areas or complex shapes are involved causing prolonged planning time. This can impact patient wait times, which are of particular concerns for a patient who has a stereotactic frame applied to their skull. Prolonged planning times can impact patient throughput as well. Different techniques and software were developed to improve the quality of the plan and standardize the treatment.

The new inverse planning tool, Lightning^®^ (Elekta AG, Stockholm, Sweden), was recently added by to the LGP software for treatment planning of Gamma Knife Radiosurgery as a fully automated inverse planner. It allows for individual or simultaneous planning of several targets and calculates the ideal dose plan based on the planning constraints decided by the user. This allows for prioritization of coverage, selectivity, low dose fall off, dose gradient in normal brain as well as dose limits on critical organs at risk (OAR).

In this paper we compare LGP optimization with the Wizard^®^ and Lightning^®^ to determine their advantages and limitations. Manual forward planning is highly user dependent and was therefore not used as a benchmark for comparison.

## Materials and methods

Wizard^®^ was used on a clinically commissioned Gamma Knife station, while the beta version of the Lightning^®^ was used on a research machine. Thirty-eight cases were compared under four different planning techniques and the results were analysed and compared according to the total time to plan (TP), number of shots (SH), coverage (C), selectivity (S), gradient index (GI), treatment delivery time (DT) and maximum dose received by an OAR. All these cases were based on real patients previously treated at our institution that presented challenges in their planning. Clinical treatments were delivered independent of this study and were not influenced by these simulations. For standardization the treatment dose and number of fractions were the same as the prescribed for those patients originally. Both Wizard^®^ and Lightning^®^ were kept in their default mode. The limit doses for the OARs were based on current clinical guidelines. We limited the cochlea’s modiolus to a maximum dose of 4.5 Gy and the optic apparatus to 8 Gy in a single session and for the 18 Gy for 3 fractions.

The first technique used was LGP with manual selection of the isocenters placement and collimator selection (4, 8 or 16mm) by the planner until coverage greater than 90% was obtained followed by Wizard^®^ optimization (M+OP). The default settings on the Wizard^®^ optimizer allow equal weights for coverage and selectivity and 0.25 weight for beamon time (BOT). Second, inverse planning (IP) with the auto-fill tool was used followed by Wizard^®^ optimization with default settings. The optimization was continued in both M+OP and IP approaches until no significant improvement was seen in the objective function displayed in the optimizer window. Third, Lightning^®^ was used without constraining for OARs (LNR). And fourth, Lightning^®^ was used with constraints for OARs (LWR). Fifteen of the 38 cases did not require constraints on specific OARs, therefore, LWR wasn’t used. The goal was to compare the optimization capabilities of both software, not necessarily obtaining a clinically deliverable plan, which can differ from a do-simetrically superior plan on occasion-based BOT and other clinical considerations. All plans on a given case were prescribed the same treatment dose, number of fractions and OAR limits and, in the first two modalities, the same isodose line (IDL) for normalization. Lightning^®^ automatically chooses a normalization IDL, and to approximate an IDL close to the other modalities would require manipulation of Lightning^®^ settings in a manner that is not intuitive and seldom used in a clinical setting.

Comparative statistics was evaluated across all patients and within each diagnostic subgroup as they can present unique planning challenges due their location, shape, and specific OARs. Pearson correlation coefficients were calculated between time to plan (TP), and lesion volume to compare each planning modality.

## Results

### General

**FIGURE 1.**
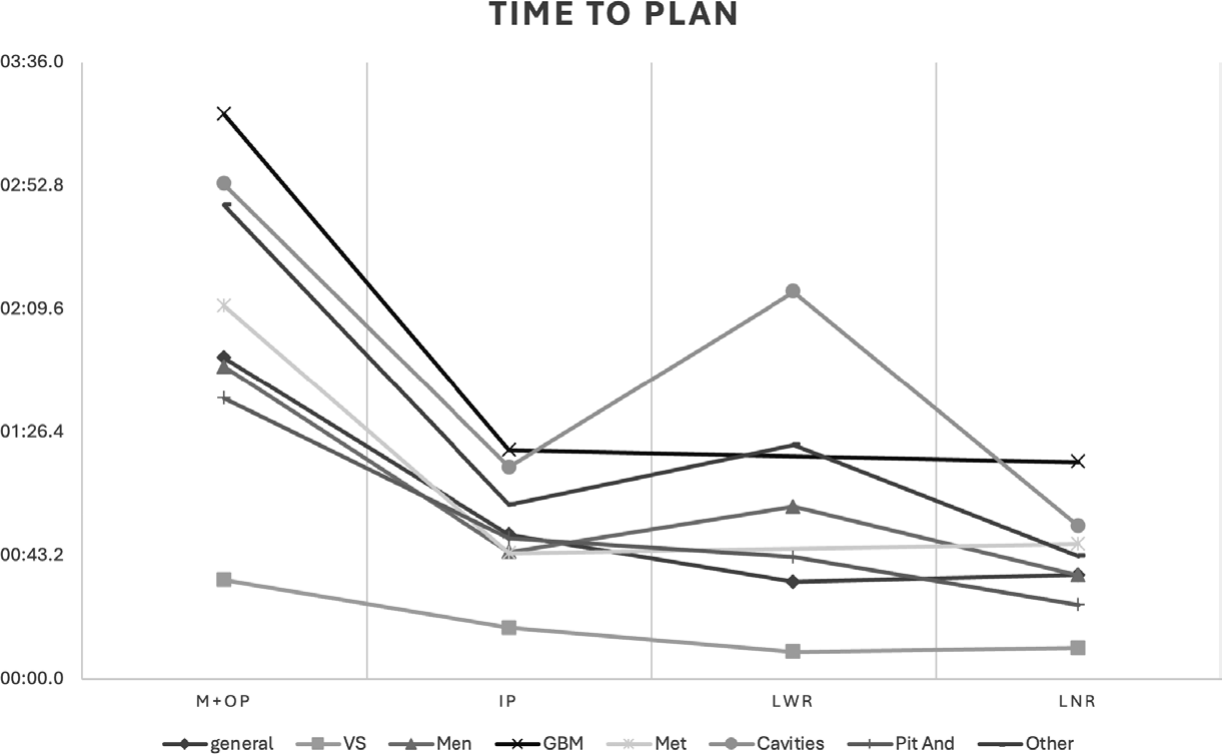
Comparison of the planning time according to each modality and pathology. GBM = glioblastoma multiform; IP = Inverse planning; LNR = Lightning^®^ with no consideration of risk structure; LWR: Lightning^®^ with consideration of risk structure; M+OP = Manual planning with optimization; Men = meningioma; Met = metastasis; Pit And = pituitary adenoma; VS = vestibular schwannoma

In the 38 case simulations, M+OP and IP were not always able to generate a plan with coverage greater than 95% but did present better selectivity. Although LWR presented a median TP shorter then LNR, this was only observed in the VS patients. LWR had greater selectivity and lower GI, but at the cost of a longer DT. (Figure 1; Table 1).

**TABLE 1.** Median results and intervals for the 38 simulation-patients. Median tumor volume was 6.356cc

General	LNR	LWR	IP	M+OP
**Time to plan (min:sec)**	00:36.4 (± 00:53.2)	00:34.1 (± 00:22.9)	00:50.6 (± 00:20.7)	01:52.6 (± 00:16.6)
**Shots**	63 (± 23.3)	69 (± 33.8)	27.5 (± 7.5)	20 (± 7.8)
**Coverage**	0.99 (± 0.14)	0.99 (± 0.32)	0.92 (± 0.43)	0.91 (± 0.27)
**Selectivity**	0.78 (± 2.1)	0.82 (± 0.69)	0.92 (± 1.93)	0.90 (± 1.78)
**Gradient Index**	2.63 (± 0.01)	2.53 (± 0.05)	2.88 (± 0.03)	3.17 (± 0.26)
**Delivery Time (min)**	46.45 (± 0.68)	49.3 (± 3.49)	52.5 (± 11.75)	47.85 (± 10.04)

1 IP = Inverse planning; LNR = Lightning^®^ with no consideration of risk structure; LWR = Lightning^®^ with consideration of risk structure; M+OP = Manual planning with optimization

The median results of the plans using Lightning^®^ and the optimization from LGP, showed that the time for planning had a reduction of 57%, coverage improvement of 8%, GI improvement of 15%, delivery time was 5% faster, selectivity decreased 12% and number of shots increased by 226%. (Table 2).

**Table 2.** Median of the parameters evaluated with Lightning^®^ (LNR and LWR) and Wizard^®^ (IP and M+OP) and their difference in percentage

	Lightning (LNR and LWR)	Wizard (M+OP and IP)	Difference
**TP (min:sec)**	00:35.3	01:21.6	-57%
**Shots**	77.5	23.75	226%
**Coverage**	0.99	0.915	8
**Selectivity**	0.8	0.91	-12%
**GI**	2.58	3.025	-15%
**DT (min)**	47.875	50.175	-5%

1 DT = delivery time; GI = gradient index; IP = Inverse planning; LNR = Lightning^®^ with no consideration of risk structure; LWR = Lightning^®^ with consideration of risk structure; M+OP = Manual planning with optimization; TP = time to plan.

### Vestibular schwannomas

**FIGURE 2.**
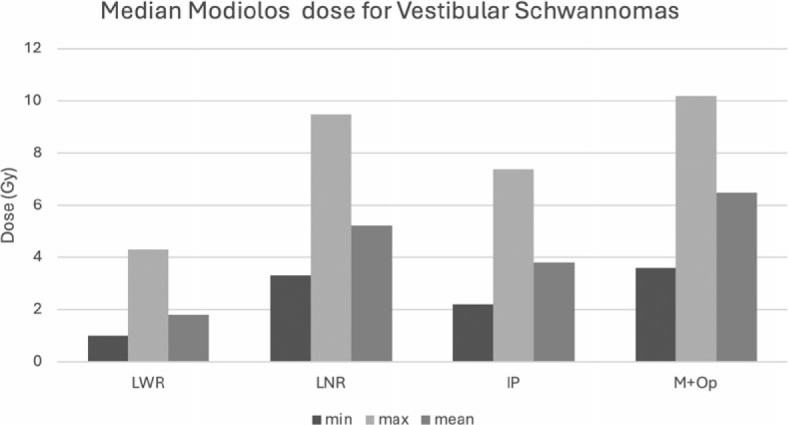
Results of the dose received by the cochlea’s Modiolos in a vestibular schwannoma plan. IP = Inverse planning; LNR = Lightning^®^ with no consideration of risk structure; LWR = Lightning^®^ with consideration of risk structure; M+OP = Manual planning with optimization

The planning results using Lightning^®^ with and without OAR had similar results, being better than the other techniques, but with more shots and longer DT. Inverse planning and manual planning had approximately 7 times less shots but resulted in compromise in coverage, 0.88 and 0.91 respectively (Table 3). OAR protection is shown in Figure 2.

**TABLE 3.** Median results and intervals for the patients with separated by pathology and planning techniques

Coverage	Vestibular schwannoma				Meningioma				Metastasis				Cavities				Pituitary Adenoma			
Meadian Volume (CC)	1.377				6.338				6.5595				11.4525				4.267			
Planning Technique	LNR	LWR	IP	M+OP	LNR	LWR	IP	M+OP	LNR	LWR	IP	M+OP	LNR	LWR	IP	M+OP	LNR	LWR	IP	M+OP
**Time to plan (min:sec)**	00:10.9 (± 00:02.2)	00:09.7 (± 00:00.8)	00:18.0 (± 00:01.2)	00:34.8 (± 00:20.9)	00:36.4 (± 01:31.7)	01:00.5 (± 01:33.8)	00:44.4 (± 00:21.9)	01:49.5 (± 01:51.9)	00:47.3 (± 00:05.9)	-	00:44.1 (± 00:02.0)	02:10.9 (± 02:28.3)	00:53.7 (± 00:59)	02:15.7 (± 00:13.8)	01:14.2 (± 00:13.6)	02:53.0 (± 01:35.9)	00:26.1 (± 00:01.3)	00:42.8 (± 00:00.9)	00:49.4 (± 00:12.5)	01:38.4 (± 00:38.0)
**Shots**	48 (± 10.81)	42 (± 6.27)	5 (± 0.46)	7 (± 3.82)	74 (± 28.24)	69 (± 35.39)	28 (± 4.44)	18 (± 10.55)	54.5 (± 2.18)	-	27 (± 0.24)	18.5 (± 0.97)	79.5 (± 29.78)	112 (± 36.03)	35.5 (± 2.58)	31 (± 3.43)	52 (± 9.53)	69 (± 9.12)	28 (± 13.9)	23 (± 6.44)
**Coverage**	0.98 (± 0.005)	0.96 (± 0.004)	0.88 (± 0.014)	0.91 (± 0.03)	0.99 (± 0.002)	0.99 (± 0.002)	0.91 (± 0.003)	0.90 (± 0.006)	0.99 (± 0.005)	-	0.91 (± 0.013)	0.915 (± 0.011)	0.99 (± 0.009)	0.99 5 (± 0.007)	0.915 (± 0.017)	0.905 (± 0.012)	0.99 (± 0.0007)	0.99 (± 0.0031)	0.92 (± 0.0005)	0.91 (± 0.0017)
**Selectivity**	0.86 (± 0.001)	0.87 (±0.002)	0.94 (± 0.077)	0.92 (±1.78)	0.81 (± 0.01)	0.83 (± 0.01)	0.92 (± 0.01)	0.89 (± 0.01)	0.74 (± 0.036)	-	0.90 (± 0.035)	0.90 (± 0.028)	0.745 (± 0.09)	0.785 (± 0.048)	0.90 (± 0.05)	0.895 (± 0.03)	0.775 (± 0.03)	0.765 (± 0.03)	0.94 (± 0.01)	0.93 (± 0.01)
**Gradient Index**	2.49 (± 0.13)	2.51 (± 0.18)	2.61 (± 0.27)	3.17 (± 0.31)	2.55 (± 0.14)	2.505 (± 0.03)	2.88 (± 0.11)	3.085 (± 0.43)	2.77 (± 0.01)	-	3.01 (± 0.13)	3.19 (± 0.18)	2.88 (± 0.29)	2.58 (± 0.28)	2.86 (± 0.34)	3.235 (± 0.63)	2.58 (± 0.056)	2.65 (± 0.043)	2.78 (± 0.199)	3.12 (± 0.005)
**Delivery Time (min)**	37.3 (± 4.19)	49.3 (± 8.87)	27.8 (± 3.92)	24 (± 1.22)	47.3 (± 1.44)	66.2 (± 0.57)	47.2 (± 6.77)	30.9 (± 10.47)	43.1 (± 7.33)	-	52.95 (± 0.06)	56.35 (± 0.61)	54.4 (± 7.18)	77.6 (± 21.48)	75.4 (± 7.66)	63.8 (± 9.92)	43.9 (± 17.58)	49 (± 19.54)	105.9 (± 0.09)	55.5 (± 7.75)

1 IP = Inverse planning; LNR: Lightning^®^ with no consideration of risk structure; LWR = Lightning^®^ with consideration of risk structure; M+OP = Manual planning with optimization.

### Meningiomas

**FIGURE 3.**
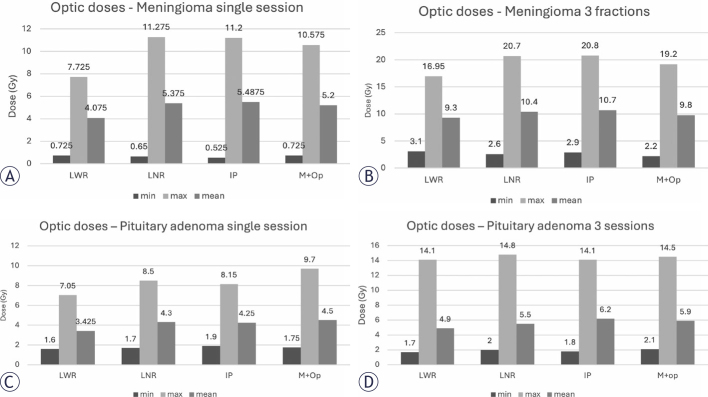
Dose received by optic structures in one **(A)** and three **(B)** sessions plans for meningiomas. Dose received by optic structures in one **(C)** and three **(D)** sessions plans for pituitary adenomas. IP = Inverse planning; LWR = Lightning^®^ with consideration of risk structure; LNR = Lightning^®^ with no consideration of risk structure; M+OP = Manual planning with optimization

IP and LNR had the quickest time, 00:44,4 and 00:36,4 respectively, but Lightning^®^ had greater coverage. Although LWR took longer to plan than inverse planning, it had better coverage and the benefit of OAR protection (Table 3). OAR protection is shown in Figure 3 A and B.

### Metastasis

From the 12 patients with metastasis in this analysis, none needed defined OARs, therefore, they were evaluated with only 3 techniques. Inverse planning was slightly quicker (44,1 sec), with 90% selectivity as the manual planning but with higher GI and DT then Lightning^®^. LNR had the best coverage, GI, and DT (Table 3).

### Surgical cavities

Ten cases of post-surgical cavities were analysed. Lightning^®^ was quicker, with better coverage, similar GI, and faster treatment time. Inverse planning and manual planning had similar coverage, selectivity, and the number of shots. Although inverse planning had best GI, it had the longest DT (Table 3)

### Pituitary adenomas

Pituitary adenomas can provide great challenges due to the tumor’s proximity to optic structures. LWR delivered a plan in 42,8 sec with 99% coverage, GI of 2.65 and DT of 49 minutes. Wizard^®^ not only took longer to plan, but also didn’t achieve adequate coverage and had longer treatment time (Table 3). OAR protection is shown in Figure 3 C and D.

### GBM

None of the GBM cases had OAR and only 3 modalities were used. Both inverse planning technics had similar TP, but Lightning^®^ had approximately 5 times the number of shots with higher DT.

### Others

On Figure 1 we can notice a close similarity on both Lightning^®^ plans with the tendency of increased number of shots but, differently from GBM, a quicker delivery time.

### Time to plan

**FIGURE 4.**
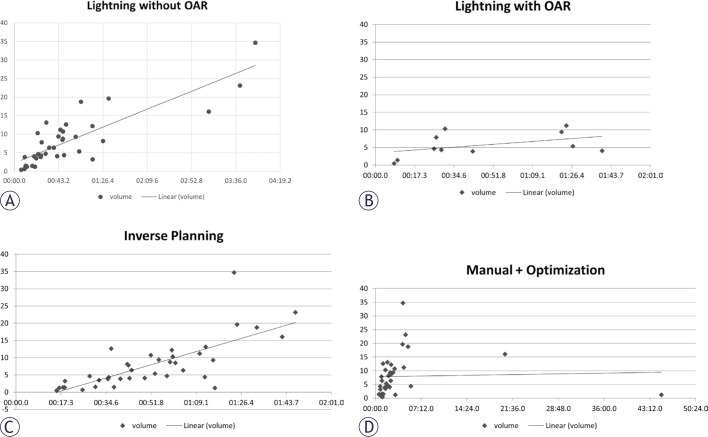
Scatter plot correlation between time to plan in minutes and tumor volume. **(A)**: Lightning^®^ with no consideration of risk structure (r = 0.84, p < 0.0001); **(B)**: Lightning® with consideration of risk structure (r = 0.45, p = 0.2); **(C)**: Inverse planning (r = 0.74, p < 0.0001); **(D)**: Manual planning with optimization (r = 0.03, p = 0.8). OAR = organs at risk

The time to plan was overall faster with Lightning^®^ as shown on Figure 1 and Table 2. An increased time to plan was noted in surgical cavities and in plans with constraints on OAR that was attributed to a greater complexity in these kind of lesions with direct implications on the calculation overhead on the planning process. Since the volume and shape can challenge the calculation engine, but only volume can be objectively quantified; Pearson correlation (PC) coefficients between tumor volume and time to plan for the four modalities were calculated being statistical significant on IP and LNR with r=.74 and .84 respectively, both p<0.0001 and M+OP (r=.03, p=0.8) and LWR (r=.45, p=0.2) not meeting statistical significancy. Their scatter plots are shown in Figure 4.

### Dose-Volume Histogram (DVH)

**FIGURE 5.**
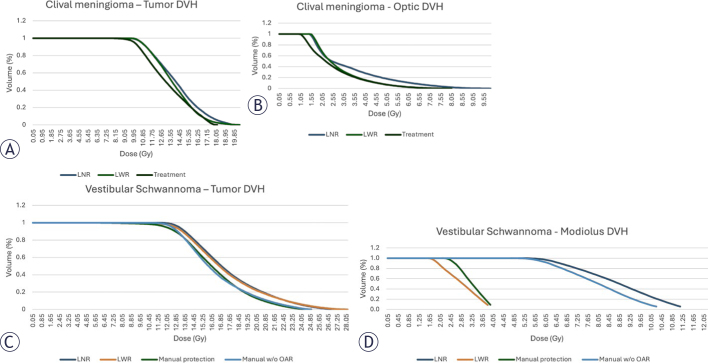
Dose-volume histogram (DVH) for clival meningioma **(A and B)** plan comparing Lightning^®^ with (LWR) and without risk (LNR) structures to a simulation of treatment planning with forward planning with **(A)** showing tumor results, and **(B)** showing the organs at risk (OAR) results. Dose-volume histogram for clival meningioma plan **(C and D)** comparing LWR and LNR to a simulation of treatment planning with forward planning with and without consideration of the OAR. **(C)** showing tumor results, and **(D)** showing the OAR results.

The presented DVH are a comparison of LWR and LNR to a deliverable forward plan. The curves show the similarity of their quality, with adequate parameters for safe treatment delivery. First case is a clival meningioma (Figure 5 A and B), where LWR had max dose to the optic of 7.25Gy while the treatment had 8 Gy. Lightning^®^ was also able to keep greater coverage. The same pattern was observed in the second case, a Vestibular Schwannoma, where LWR had greater coverage and greater Modiolus protection (Figure 5 C and D).

### Discussion

The Wizard^®^ function was introduced in LGP version 5.34 back in 2000, marking it as the first inverse planning tool available for Gamma Knife in the market. However, this early version was limited by its simplistic approach to solving a few objective functions to find the lowest penalty solution, and it was prone to getting stuck in a subpar solution (local minima) as it was not modelled as a convex problem. It did not adequately account for organs at risk (OARs). Adoption of the Gradient Index concept, leading to the development of a more sophisticated inverse planning tool in LGP version 10 (2010). This updated software promised to enhance clinical workflows, especially for planning larger, spherical targets in non-critical areas. Because the objectives use relative isodoses (instead of absolute doses) and that the positions of the shots can change, the optimization problem even in this version of the Wizard^®^ remained inherently difficult (nonconvex), unable to manage multiple targets and to enforce criteria such as the maximum dose to an OAR.^1^ Nonetheless, for more intricate targets, manual planning remains the preferred approach, especially among expert planners.^2^

Lightning^®^ models the inverse planning as a convex problem which is both faster and less prone to getting stuck in sub-optimal solutions.^1^ Wizard^®^ used repositioning, relative weight, and collimator settings inside a predetermined isodose line and could delete unnecessary shots to reach the best solution. Different settings were used to penalize according to coverage, selectivity, gradient and delivery time depending on the planner’s objective which can limit the quality of the plan 1 but, when used properly by experienced planners, it can improve.^3^ Also, a selection of fixed collimators and fixed position can be set but limiting the field delineation.

Lightning^®^ achieves convexity by dividing the planning into: isocenter placement, optimization, and sequencing. The isocenters chosen in the first phase remain fixed throughout the rest of the planning. In the optimization phase, competing objectives are combined as a weighted sum. By altering weights, it is possible to explore achievable trade-offs. Possible objectives include dose to target, sparing of OARs, and a new – highly efficient – Beam on Time (BOT) penalization. During the optimization, times for each sector and collimator are allowed to vary independently. In the sequencing phase, these times are converted into deliverable shots. Altogether this algorithm tries to find the best configuration based on the given dose, defines the best isodose line and converts the “dose cloud” into shots. It is also able to plan multiple lesions simultaneously while considering their dose contributions, differently from Wizard^®^ that is uncapable of doing these actions. The settings can be penalized to BOT and ‘lower acceptable dose’, that can be used to push for a desired isodose line by the planner.

In this beta version the number of isocenters was 3 times higher. Two patterns were observed. First, high weighted shots were surrounded by several low weight shots that helped a better delineation of the dose in complex-shaped targets. Second, overlapping shots with different collimator settings and weights, not only helped the dose delineation, but also helped decrease delivery time and allows higher IDL with appropriate fall off of the dose without jeopardizing the V12.^4^ The ability to deliver a high conformal plan in less time would diminish the ability of the tumor cells to repair^5^ affecting tumor control. Decreasing the time that the patient waits to be treated and shortening the delivery time directly impacts the patient’s tolerance and the daily dynamics of the department.

PC showed that IP and LNR had good positive correlation of target volume with planning time, but the weaking of the correlation presented with LWR can be explained by necessity to shape the dose for maximal OAR protection. The low number of shots in manual plans was perhaps an explanation of a lack of direct correlation between TP and lesion volume in the M+OP group.

Planning quality discrepancies are related to the experience of the planner and can be minimized by Lightning^®^.^6^ New planners and new centers can use this software as a learning tool to improve their skills in planning, especially when OAR places limitation in keeping adequate coverage. This allows a standardization of the plans, leading to better evaluation of the treatment in multi-center studies.

The preservation of function must be considered during planning. For this reason, the amount of dose received by important structures like the cochlea/modiolus, and the optic structures must be quantified. Lightning^®^ can plan according to a planner-specified maximum OAR tolerance dose. In the previous version of the LGP, OAR should be protected with a new definition of the shots by the planner or by the “dynamic shaping” option. In this analysis, this tool was not used. Different software solutions have been proposed with the purpose of increase IP efficacy^2,7,8,9^ each one achieving different results, but not always being successful in improving OAR protection and negatively impacting coverage and DT, which can be an issue since the biological effective dose for ultra-shortterm repair will be compromised.^5^ LWR was able to maintain adequate coverage while protecting the OAR in all tested plans while the other modalities only achieved protection for optic structures in pituitary tumors treated in 3 fractions as demonstrated in Figure 4, which is likely attributable to the anatomy of the cases and dose prescribed, not the optimization capacity.

## Conclusions

The pursuit of the perfect plan for a tumor treatment can have many challenges and achieving a proper coverage, selectivity, gradient index, and delivery time can be time consuming, while sacrificing one of these factors can lead to compromise of the plan. Practice allied to understanding of how the optimization tool works and how to adjust its settings can overcome this complexity and improve the quality of a plan and the confidence that the best solution is being offered to the patient. This is especially beneficial for new centers to understand the potentials in making complex plans and to improve their learning curve. Lightning^®^ allowed us to quickly create several plans, improving complex cases and optimizing to single or multiple session, tailoring the final plan to the patient’s needs.

The addition of Lighting^®^ is welcomed for experienced and new Gamma Knife centers allowing complex and multiple tumors to be planned quickly, with shorter delivery time and good conformability, leading to better tolerance by the patients, a more dynamic daily routine, increase in the number of daily treatments and, by optimizing the delivery time, have a radiobiological advantage by impacting the intra-fraction repair.
